# Effects of Covid-19 on male reproductive system

**DOI:** 10.1590/S1677-5538.IBJU.2021.99.04

**Published:** 2020-11-18

**Authors:** Matheus Ferreira Gröner, Renata Cristina de Carvalho, Jacqueline Camillo, Paulo Roberto Abrão Ferreira, Renato Fraietta

**Affiliations:** 1 Universidade Federal de São Paulo Disciplina de Urologia Departamento de Cirurgia São PauloSP Brasil Departamento de Cirurgia, Disciplina de Urologia, Universidade Federal de São Paulo - UNIFESP, São Paulo, SP, Brasil; 2 Universidade Federal de São Paulo Disciplina de Infectologia Departamento de Medicina São PauloSP Brasil Departamento de Medicina, Disciplina de Infectologia, Universidade Federal de São Paulo - UNIFESP, São Paulo, SP, Brasil

**Keywords:** severe acute respiratory syndrome coronavirus 2 [Supplementary Concept], COVID-19 diagnostic testing [Supplementary Concept], Viruses, Reproduction

## INTRODUCTION

The world is currently facing a pandemic resulted of a Coronaviridae family virus global spread declared by the World Health Organization (WHO) as public health emergency (1, 2). The first coronaviruses with human infection properties were isolated in 1937, but it was not until 1965 that this agent received its name based on its microscopic crown-shaped structure (3). Due to emergence of this virus and the new wave of infections, worldwide research is focused in better understanding its characteristics in order to outline current and effective ways of fighting against it (1).

Out of the six types of virus from the Coronaviridae Family, the Severe Acute Respiratory Syndrome Coronavirus 2 (SARS-CoV-2) is the one responsible for the Coronavirus Disease 2019 (COVID-19) (4); this virus has 80% if its gene structure identical to the SARS-CoV, responsible for the SARS pandemic in 2002 (5).

There is a consensus that the main form of contagion of this disease is from person to person through droplets derived from sneezing or coughing (6) and that the gold standard diagnosis tool is the real-time reverse transcription polymerase chain reaction (RT-PCR) of samples collected by nasopharyngeal and oropharyngeal swab (7). Despite that, the virus has already been isolated in urine (8), feces (8), conjunctiva (9) and saliva (10) from infected patients. Hence, could the virus also be found in the semen of infected males?

There are more than 27 viruses (HIV, mumps, zika, among others) that can be found in semen, which indicates the virus potential to reach organs of the male reproductive system (11–14). Beyond the transmissibility matter, previous studies indicate that, when present in semen, some virus can affect the male fertile potential (15); therefore, it is important to investigate SARS-CoV-2 presence in semen of infected men while also evaluating possible changes on their fertile potential.

In view of the genetic similarity between the etiological agents of SARS and COVID-19, it is possible to infer the probable effects of SARS-CoV-2 on the male reproductive system based on previous studies on SARS-CoV. There are no reports on the presence of SARS-CoV in semen in patients with SARS, however there were descriptions of orchitis and deleterious effects on testicular tissue in autopsies (16, 17) with confirmation of the virus presence in the testicles (18).

Moreover, the mechanism of cellular infection of SARS-CoV-2 is similar to SARS-CoV, due to the link between the viral Spike (S) protein and the Angiotensin converting enzymes 2 (ACE2) cell receptor (19–21). Previous studies have shown the high concentration of these receptors in the germ and somatic cells of the testicular tissue (22). This fact may indicate the testicles tissue vulnerability to contamination by this new virus, reinforcing the importance of monitoring the reproductive function in infected patients.

The purpose of this narrative review is to evaluate published evidence on possible effects of COVID-19 on male reproductive system.

## MATERIALS AND METHODS

A narrative review was done with the aim to identify all relevant studies on SARS-CoV-2 and male reproductive system. We performed a search on Pubmed platform using keywords such as “covid 19”, “SARS-CoV-2”, “pandemic”, “infection” and “virus” added to the Boolean operators “AND”, “OR” and combined with others terms such as “cell receptors”, “semen”, “gonadal function” and “testicles”. No temporal limits were set for the database searches as the topic is recent and little published literature is available. Only articles written in English were considered.

### Cellular receptors associated with the infectious process

Due to the similarity related to the infection pathogenesis between SARS-CoV and SARS-CoV-2, a recent report has already described the importance of the ACE2 cells receptor for the initial binding between virus and cell, which initiates the cell fusion and invasion process (21). As result, several studies have demonstrated the ACE2 receptor concentrations in different human tissues, predicting the possibility of infection in these systems. For this review, we limited our analysis to studies that evaluated the tissues of the male reproductive system.

**Figure 1:**
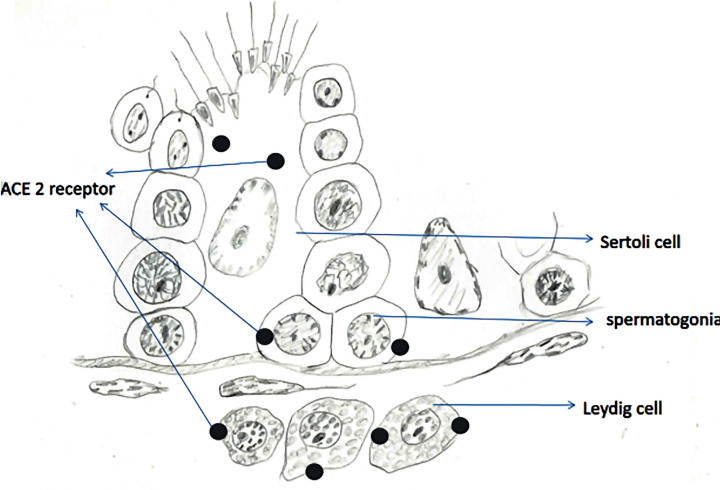
Scheme depicting the location of ACE2 receptor, a target for SARS-CoV-2 infection, in testicular cells.

Different methods can be applied to investigate the presence of receptors in a tissue, but most the reviewed studies have done their analysis through bioinformatics associated with gene sequencing of RNA expression (23). The results have demonstrated ACE2 highly expressed in Leydig cells and cells of the seminiferous tubules (24), besides high expression in germ cells (25, 26) (Figure-1). These findings were also confirmed by another study that demonstrated that the testicular tissue has the highest concentration of ACE2 receptors when compared to other human tissues, higher even than the lung tissue, main target of the disease (27). This study still performed immunohistochemistry analysis that showed high ACE2 expression in sperm and Leydig cells, moderate expression in seminiferous vesicle glands and low expression in the prostate and bladder (27).

These results provide evidences that the testicles are vulnerable to infection by SARS-CoV-2, however, with such a concentration of receptors in the testicular tissue, why is the infection not clinically evident in this system? More recent studies have shown that as important as the presence of ACE2 receptor is the presence of a transmembrane protease named Transmembrane Serine Protease 2 (TMPRSS2). This protease is responsible for assisting the breakdown of the viral S protein favoring its fusion and invasion into the cell (28). When assessing the ACE2 receptors and TMPRSS2 proteases co-expression, a low (29) or extremely rare (30) expression was observed in testicular tissue, in contrast to the high co-expression identified in pneumocytes and nasal epithelial cells (31), which explains the high frequency of respiratory symptoms in COVID-19. This high co-expression was also observed in the ileum, heart and kidney (32), which may be related to the gastrointestinal symptoms described and the high rates of heart and kidney complications associated to the disease (33–35).

According to these findings, the infection by SARS-CoV-2 in the male reproductive system is unlikely to occur. However, it is important to note that virus can find other ways to infect the cell besides ACE2 receptors and TMPRSS2 proteases (36, 37), nevertheless, the RNA sequencing method for ACE2 and TMPRSS2 evaluation is also subject to bias and errors. In that sense, the true effects of the virus on the male reproductive system must be further evaluated through clinical studies.

### SARS-CoV-2 presence in semen and other secretions

The initial clinical studies evaluating the presence of SARS-CoV-2 virus in semen of infected patients using RT-PCR tests have not detected virus presence in the samples. These studies, however, evaluated a small number of patients (between 12 and 34 individuals) and most of them were in recovery periods from the disease, on average 30 days after the disease onset (29, 38). Despite this, orchialgia complaints were noted in 19% of the patients (29), which could lead us to infer probable testicular involvement in the disease process but not all patients in the study had a comprehensive genitourinary examination which limits these result interpretations.

A subsequent study analyzed semen from 38 inpatients diagnosed with COVID-19, 15 patients were in the acute phase and 23 were already recovered from the disease. Viruses were found in semen of 6 patients, 4 (15.8%) who were in the acute phase and 2 (8.7%) who were in the recovery phase (2 and 3 days of recovery) (39). This was the first study that demonstrated the presence of the virus in semen.

When considering the nasopharyngeal and oropharyngeal secretion RT-PCR, the peak of sensitivity occurs at the symptoms onset with rare cases maintaining positive results after 21 days of infection, a pattern different from the tracheal secretion that shows the peak of sensitivity at the 11th day of infection and the positivity remains longer (40, 41). These indicates a probable window of virus exposure that can vary according to which secretion that is been evaluated; the study shows that the presence of virus in semen is more evident in the acute phase of disease beginning to identify the window of positivity in this secretion.

A higher and longer level of viral load is observed in severe cases when compared to patients with milder symptoms (41). Thus, another point to be considered is that hospitalized patients with potentially severe cases and greater viremia were selected for the study that identified virus presence in the semen, differently from previous studies with negative results that sole evaluated recovered individuals.

In order to validate these results, a prospective follow-up of those patients would be important to understand for how long the virus remains in the semen. Moreover, specific studies to analyze the possibility of viral transmissibility by this secretion could enhance the impact of the infection in the male reproductive system.

Another study yet analyzed prostate secretion in the urine after prostate massage. Viral research was negative in all 23 evaluated patients, even with 75% of them in the acute phase of the disease (42).

### Gonadal function of patients with COVID-19

Only one of the reviewed studies evaluated gonadal function in COVID-19 patients using a hormonal profile. When compared to healthy individuals, infected patients showed increased LH levels and decreased Testosterone: LH ratio, indicating a probable initial gonadotoxic effect (43). The study evaluated 81 patients classified with moderate or severe disease, defined as presence of fever and cough associated with radiological changes, which could have biased the comparison with healthy individuals. Feverish conditions are known to potentially alter gonadal function (44, 45) and thus, the changes observed in the study could be related to the fever symptoms and not specifically to the COVID-19 infection.

There are no data in the literature regarding the fertile potential of men with COVID-19 as none of the studies performed a seminal analysis. Different viral infections can have a direct effect on gonadal function, such as mumps infection (46) and other viruses (15). Thus, it is important to prospectively analyze COVID-19 patients in order to investigate gonadal dysfunction associated with the condition.

### Future perspectives

SARS-CoV-2 has already been found in semen of infected patients, but several questions remain unanswered: Can SARS-CoV2 virus be transmitted through semen?

Can SARS-CoV-2 infection lead to gonadal dysfunction or fertile potential loss?

Are those changes reversible after disease recovery? Further prospective studies are needed to specifically cover these points.

## CONCLUSIONS

As any emergent disease, there are more suspicions and hypotheses than certainties in terms of COVID-19 effects on male reproductive system. Numerous studies have been carried out to better understand the disease and its short and long-term repercussions on health status. As demonstrated in other viral diseases, involvement of the male reproductive system is a possibility and it may reveal a new route of transmission and/or repercussions on its functions. The virus has already been found in the semen of infected patients but its impacts on male reproductive health have yet to be further investigated.

## ABBREVIATIONS

SARS-CoV-2: Severe Acute Respiratory Syndrome Coronavirus 2
COVID-19: Coronavirus Disease 2019
SARS-CoV: Severe Acute Respiratory Syndrome Coronavirus
SARS: Severe Acute Respiratory Syndrome
RT-PCR: Real-Time reverse transcription Polymerase Chain Reaction
HIV: Human Immunodeficiency Virus
S: Spike protein
ACE2: Angiotensin converting enzymes 2
RNA: Ribonucleic acid
TMPRSS2: Transmembrane Serine Protease 2
LH: Luteinizing Hormone
